# Alcohol Medication

**Published:** 1914-07

**Authors:** 


**Alcohol Medication.** In reply to an editorial in the July, 1913, number of American Medicine, Critic and Guide, May, 1914, reprinted from American Medicine, Abraham Jacobi breaks a lance for the use of alcohol in cases of sepsis. He tells how he has tried, in his sixty years of practice, all the new remedies recommended for these conditions only to return to alcohol, and he refutes Ewald's statement that "all theories to the effect that it is to be classed as a stimulant are about exploded," by quoting some of his personal experiences of desperate cases in which alcohol has seemed to him to be of great benefit.—C. G. L-W.

